# Clinical Relevance of the Tumor Location-Modified Laurén Classification System for Gastric Cancer in a Western Population

**DOI:** 10.1245/s10434-021-11252-y

**Published:** 2022-01-18

**Authors:** J. L. Moore, A. R. Davies, A. Santaolalla, M. Van Hemelrijck, N. Maisey, J. Lagergren, J. A. Gossage, M. Kelly, C. R. Baker, on behalf of the Guy’s and St. Thomas’ Esophago-gastric Research Group, A. Jacques, N. Griffin, V. Goh, S. Ngan, A. Lumsden, K. Owczarczyk, A. Qureshi, H. Deere, M. Green, F. Chang, U. Mahadeva, B. Gill-Barman, S. George, J. Meenan, M. Hill, J. Waters, M. Cominos, O. Hynes, G. Tham, R. K. Bott, J. M. Dunn, S. S. Zeki

**Affiliations:** 1 Department of Upper Gastrointestinal and General Surgery, St. Thomas’ Hospital, London, UK; 2 School of Cancer and Pharmaceutical Sciences, King’s College, London, UK; 3 Translational Oncology and Urology Research (TOUR), School of Cancer and Pharmaceutical Sciences, King’s College, London, UK; 4 Department of Medical Oncology, St. Thomas’ Hospital, London, UK; 5 Department of Molecular Medicine and Surgery, Karolinska Institutet, Stockholm, Sweden

## Abstract

**Background:**

The Tumor Location-Modified Laurén Classification (MLC) system combines Laurén histologic subtype and anatomic tumor location. It divides gastric tumors into proximal non-diffuse (PND), distal non-diffuse (DND), and diffuse (D) types. The optimum classification of patients with Laurén mixed tumors in this system is not clear due to its grouping with both diffuse and non-diffuse types in previous studies. The clinical relevance of the MLC in a Western population has not been examined.

**Methods:**

A cohort study investigated 404 patients who underwent gastrectomy for gastric adenocarcinoma between 2005 and 2020. The classification of Laurén mixed tumors was evaluated using receiver operating characteristic (ROC) curve analysis and comparison of clinicopathologic characteristics (chi-square). Survival analysis was performed using multivariable Cox regression.

**Results:**

The ROC curve analysis demonstrated a slightly higher area under the curve value for predicting survival when Laurén mixed tumors were grouped with intestinal-type rather than diffuse-type tumors (0.58 vs 0.57). Survival, tumor recurrence, and resection margin positivity in mixed tumors also was more similar to intestinal type. Distal non-diffuse tumors had the best 5-year survival (DND 64.7 % vs PND 56.1 % vs diffuse 45.1 %; *p* = 0.006) and were least likely to have recurrence (DND 27.0 % vs PND 34.3 % vs diffuse 48.3 %; *p* = 0.001). Multivariable analysis demonstrated that MLC was an independent prognostic factor for survival (PND: hazard ratio [HR], 1.64; 95 % confidence interval [CI], 1.16–2.32 vs diffuse: HR, 2.20; 95 % CI, 1.56–3.09)

**Conclusions:**

The MLC was an independent prognostic marker in this Western cohort of patients with gastric adenocarcinoma. The patients with PND and D tumors had worse survival than those with DND tumors.

**Supplementary Information:**

The online version contains supplementary material available at 10.1245/s10434-021-11252-y.

Gastric cancer is the fourth most common cause of cancer-related mortality worldwide, accounting for nearly 800,000 deaths in 2020.^1^ Introduced in 1965, the Laurén classification system is the most widely used histologic classification of gastric cancer. It describes three subtypes (intestinal, diffuse, and mixed), which demonstrate distinct clinicopathologic characteristics. The intestinal type is characterized by cohesive cells arranged into glandular formations. It is commonly associated with intestinal metaplasia, chronic inflammation and *Helicobacter* infection.^2^ In the diffuse type, tumor cells lack adhesion, infiltrate the stroma as single cells or small subgroups, and commonly form signet ring cells. It often is associated with younger female patients and a poorer prognosis.^2–5^ The mixed type is characterized as a non-homogeneous mixture of the intestinal and diffuse types. Anatomic tumor location also has been shown to affect prognosis, with tumors located in the proximal third and gastric cardia demonstrating poorer survival than those in the middle or distal stomach.^6–8^

A recent article proposed a Tumor Location-Modified Laurén Classification (MLC) system combining Laurén pathologic type with anatomic location.^9^ This system describes three distinct subtypes of gastric cancer: proximal non-diffuse type (PND), distal non-diffuse type (DND), and diffuse type (D). The MLC system is supported by differences in RNA expression profiles between subtypes.^9^

Two previous studies have explored the clinical relevance of the MLC system. One study demonstrated its positive prognostic ability for patients with early gastric adenocarcinoma in a Korean population,^10^ and a study from China found the MLC was a more reliable prognostic marker than the original Laurén classification.^11^

In the proposed MLC, patients with Laurén mixed-type histology are grouped together with Laurén intestinal type, (i.e. in the DND or PND groups), and the diffuse group is exclusively made up of Laurén diffuse-type tumors. However, another study included Laurén mixed-type tumors with the diffuse group, with the authors providing no explicit rationale for this change in classification.^10^

Laurén mixed-type tumors have been shown to demonstrate unique biologic and clinical behavior and make up approximately 20 % of gastric adenocarcinomas.^3, 12–14^ It is therefore important to understand where they are best classified in the MLC.

This study aimed first to establish where patients with Laurén mixed-type tumors are best classified within the MLC system when it is used to predict survival. The second aim was to determine whether the MLC independently predicts survival in a Western cohort of patients with resectable gastric adenocarcinoma when adjusted for confounding factors.

## Materials and Methods

### Patients

This cohort study was based on a prospectively maintained database of consecutive gastric resections performed at Guy’s and St. Thomas’ Hospital, London in the United Kingdom. The study included all patients who underwent gastrectomy with curative intent for histologically confirmed gastric adenocarcinoma between February 2005 and February 2020, with follow-up assessment until February 2021.

Gastrectomy (total or subtotal) with D2 lymphadenectomy was performed by either an open or laparoscopic approach for all the patients. Tumors were staged using American Joint Committee on Cancer (AJCC) TNM version 7. The primary outcome measure was overall survival. The secondary outcomes were disease-free survival, prognostic ability, and optimum categorization of the MLC.

### The Modified Laurén Classification

The MLC system divided gastric cancer into three subtypes:^9^ (1) PND tumors are those whose bulk (>80 %) is located in the gastric cardia but may extend up to the gastroesophageal junction and a small portion of the distal esophagus. Their pattern of tumor infiltration should not be entirely diffuse (i.e., Laurén intestinal or mixed type). (2) DND tumors are those whose bulk is located in the distal stomach and may extend up to the mid body or down to the pylorus. Their dominant pattern is Laurén intestinal type, but also may include Laurén mixed type. (3) Diffuse tumors may occur anywhere in the stomach. Their pattern of infiltration is entirely diffuse without any component of gland-forming intestinal-type carcinoma. A variation of this classification included Laurén mixed type with diffuse rather than non-diffuse (DND/PND) tumors.^10^

### Statistical Analysis

To identify where patients with Laurén mixed type tumors were best placed within the MLC system, the predictive accuracy of different models was determined using receiver operating characteristic (ROC) curve analysis to calculate area under the curve (AUC). An AUC of 1.0 indicates perfect predictive ability, and an AUC of 0.5 indicates no ability. In the first model, the patients with mixed-type histology were grouped with non-diffuse type (DND/PND) tumors as per the MLC system described by Shah et al.^9^ (Shah MLC). In the second model, they were grouped with diffuse-type tumors as per the modified MLC system used by Choi et al.^10^ (Choi MLC).

A ROC analysis was performed for uni- and multivariate models including relevant covariables (defined later). These results determined how Laurén mixed tumors were categorized for survival analysis. Further ROC analysis also was performed to compare the predictive ability of the MLC with that of the original Laurén classification. Clinicopathologic characteristics of the patients categorized by the MLC and original Laurén classification were compared using the chi-square test.

Overall survival was defined as the time from surgery to death or date of last outpatient department visit. Disease-free survival was defined as the time from surgery to cancer recurrence, death, or date of last outpatient department visit. Survival curves were calculated using the Kaplan-Meier method, with subgroups compared using the log-rank test. Unadjusted and multivariable survival analyses were performed using Cox proportional hazards regression with adjustment for age, sex, pT stage (pT1–2, pT3–4), pN stage (pN0, pN1, pN2–3), differentiation (well/moderate, poor), lymphovascular invasion (LVI), and resection margin status. These confounders were defined based on directed acyclic graphs.^15^

All *p* values lower than 0.05 were considered to be statistically significant. Statistical analyses were performed using SAS software (version 9; SAS Institute Inc., Cary, NC, USA) and GraphPad Prism 9 (GraphPad Software Inc., San Diego, CA, USA).

## Results

### Classification of Laurén Mixed-Type Tumors

From the database, 404 eligible patients were identified, including 270 males (66.8 %) and 134 females (33.2 %). The mean age was 66.7 years (range, 26–93 years). The tumor types comprised 229 Laurén intestinal type, 84 Laurén mixed type, and 91 Laurén diffuse type tumors. A clinicopathologic comparison between Laurén subtypes (Table S1) demonstrated that the mixed-type tumors were more similar to the intestinal type in some domains (lower resection margin positivity, higher rates of LVI, better survival, and less tumor recurrence). However, they were more comparable with the diffuse type in other domains (more advanced pT and pN stage and lower rates of HER2 positivity).

**Fig. 1:**
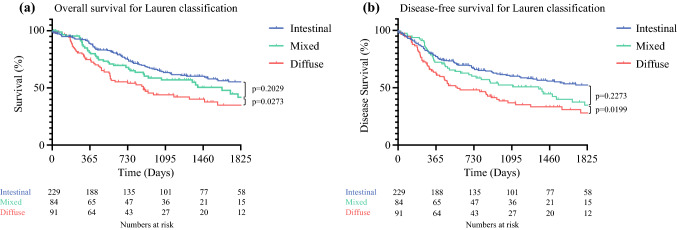
Kaplan-Meier survival analysis of the Laurén classification for **a** overall survival and **b** disease-free survival. The significance of the difference between survival curves was calculated using the log-rank test

To identify where Laurén mixed-type tumors were best placed within the MLC system, ROC curve analysis was performed, providing AUC values for overall and disease-free survival respectively. The AUC values for the univariate Shah MLC model were relatively low (0.58 and 0.59), but were higher than for the Choi MLC model (0.57 and 0.58). A separate ROC curve analysis for the Laurén classification gave lower AUC values again (0.56 and 0.57). Kaplan-Meier survival analysis (Fig. 1) demonstrated that survival curves for Laurén mixed-type tumors were more similar to those for Laurén intestinal-type tumors (*p* = 0.20) than for diffuse-type tumorrs (*p =* 0.027, log-rank). Because these results suggested that grouping of Laurén mixed-type tumors with non-diffuse-type tumors (DND/PND) was superior when the MLC was used to predict survival, this categorization was used for subsequent analysis.

### Patient and Treatment Characteristics

The 404 study patients comprised 215 patients with DND tumors, 98 patients with PND tumors, and 91 patients with diffuse tumors (Table 1). The patients with diffuse tumors were younger and more likely to be female. The patients with PND tumors were more likely to be overweight (body mass index [BMI] ≥25) than those with DND tumors.

**Table 1:** Demographics, treatment characteristics, and survival comparison between the tumor location- modified Laurén classification subtypes

Variable	Distal non-diffuse (*n =*215) *n* (%)	Proximal non-diffuse (*n =* 98) *n* (%)	Diffuse (*n =* 91) *n* (%)	(*p* Value)
Age at operation: years (range)	70 (34–93)	68 (34–87)	65 (26–83)	DND vs PND (0.53) DND vs D (0.004) PND vs D (0.047)
Sex				
Male	146 (67.9)	76 (77.6)	48 (52.7)	DND vs PND (0.08) DND vs D (0.01) PND vs D(<0.001)
Female	69 (32.1)	22 (22.4)	43 (47.3)	
BMI				
<25	60 (41.4)	18 (26.9)	25 (41.0)	DND vs PND (0.04) DND vs D (0.97) PND vs D (0.09)
≥25	85 (58.6)	49 (73.1)	36 (59.0)	
Not recorded	*70*	*31*	*30*	
Neoadjuvant treatment				
Yes	77 (35.8)	76 (77.6)	51 (56.0)	DND vs PND (<0.001) DND vs D (0.001) PND vs D (0.002)
No	138 (64.2)	22 (22.4)	40 (44.0)	
Operation				
Total gastrectomy	23 (10.7)	97 (99.0)	38 (41.8)	N/A
Subtotal gastrectomy	192 (89.3)	0 (0)	53 (58.2)	
Other	0 (0)	1 (1)	0 (0)	
Recurrence				
No recurrence	157 (73.0)	64 (65.3)	47 (51.7)	Overall (0.001) DND vs PND (0.17) DND vs D (<0.001) PND vs D (0.06
Recurrence	58 (27.0)	34 (34.7)	44 (48.3)	
5-Year survival				
Alive	139 (64.7)	55 (56.1)	41 (45.1)	Overall (0.006) DND vs PND (0.15) DND vs D (0.002) PND vs D (0.13)
Not alive	76 (35.3)	43 (43.9)	50 (54.9)	
Recurrence pattern				
No recurrence	157 (73.0)	64 (65.3)	47 (51.7)	DND vs PND (0.29) DND vs D (<0.001) PND vs D (0.002)
Local recurrence	10 (4.6)	2 (2.0)	6 (6.6)	
Systemic recurrence^a^	23 (10.8)	17 (17.4)	6 (6.6)	
Peritoneal recurrence	10 (4.6)	7 (7.1)	20 (22.0)	
Mixed recurrence^b^	15 (7.0)	8 (8.2)	12 (13.1)	

*DND* distal non-diffuse gastric tumor, *PND* proximal non-diffuse gastric tumor, *D* diffuse gastric tumor, *BMI* body mass index

^a^Systemic recurrence is a hematogenous or distant lymph node metastatic recurrence.

^b^Mixed recurrence is any combination of recurrence patterns.

All the patients with PND tumors underwent total gastrectomy except for one patient who underwent laparoscopic partial gastrectomy of the gastric fundus for a T1bN0 tumor. Most of the patients with DND tumors underwent subtotal gastrectomy (89.3 %, 192/215), with the remainder undergoing total gastrectomy. The patients with DND tumors who underwent total gastrectomy did so because of bulky tumors extending to the mid body of the stomach (*n =* 18) or because high-grade dysplasia was identified in the proximal stomach (*n =* 5). Slightly more than half of the patients with diffuse tumors underwent subtotal gastrectomy (58.2 %, 53/91), with the remainder undergoing total gastrectomy. The patients with DND tumors were the least likely and the patients with PND tumors the most likely to receive neoadjuvant chemotherapy (DND 35.8 % vs PND 77.6 % vs D 56 %).

### Pathologic Characteristics

Diffuse and PND tumors were more likely to be locally advanced (stage pT3-4) and have lymph node metastases (Table 2). By definition, all diffuse tumors were poorly differentiated. There was no major difference in differentiation between the PND and DND tumors. The diffuse tumors had lower rates of human epidermal growth factor receptor 2 (HER2) positivity and LVI. The diffuse tumors were more likely to have a positive longitudinal resection margin (DND 3.3 % vs PND 6.1 % vs D 27.5 %). Further analysis of the resection margins in this group (Table S2) showed similar positive margin rates for subtotal and total gastrectomy. There was a trend toward higher rates of proximal margin positivity in total gastrectomy and higher rates of distal margin positivity in subtotal gastrectomy, although this difference did not reach statistical significance. The overall positive longitudinal resection margin rate for the entire cohort was 9.4 %.

**Table 2:** Comparison of pathologic characteristics between the Tumor Location-Modified Laurén Classification subtypes

Variable	Distal non-diffuse (*n =* 215) *n* (%)	Proximal non-diffuse (*n =* 98) *n* (%)	Diffuse (*n =* 91) *n* (%)	(*p* Value)
pT stage				
pT0-2	117 (54.4)	35 (35.7)	26 (28.6)	DND vs PND (0.002) DND vs D (<0.001) PND vs D (0.29)
pT3-4	98 (45.6)	63 (64.3)	65 (71.4)	
pN stage				
pN0	99 (46.0)	39 (39.8)	29 (31.9)	DND vs PND (0.49) DND vs D (0.01) PND vs D (0.05)
pN1	42 (19.5)	24 (24.5)	14 (15.4)	
pN2-3	74 (34.5)	35 (35.7)	48 (52.7)	
Differentiation				
Well/moderate	94 (43.7)	52 (53.1)	0 (0)	DND vs PND (0.12) DND vs D (<0.001) PND vs D (<0.001)
Poor	121 (56.3)	46 (46.9)	91 (100)	
Laurén type				
Intestinal	152 (70.7)	77 (78.6)	N/A	N/A
Mixed	63 (29.3)	21 (21.4)	N/A	
Diffuse	N/A	N/A	91 (100)	
LVI				
No	81 (37.7)	31 (31.6)	44 (48.4)	DND vs PND (0.30) DND vs D (0.08) PND vs D (0.02)
Yes	134 (62.3)	67 (68.4)	47 (51.6)	
HER2 status				
Positive	24 (17.5)	9 (16.1)	2 (3.6)	DND vs PND (0.81) DND vs D (0.01) PND vs D (0.05)
Negative	113 (82.5)	47 (83.9)	54 (96.4)	
Not recorded	78	42	35	
Resection margin				
R0	208 (96.7)	92 (93.9)	66 (72.5)	DND vs PND (0.24) DND vs D (<0.001) PND vs D (<0.001)
R1	7 (3.3)	6 (6.1)	25 (27.5)	

DND, distal non-diffuse gastric tumor; PND, proximal non-diffuse gastric tumor; D, diffuse gastric tumor; N/A, Not applicable; LVI, lymphovascular invasion; HER2, human epidermal growth factor receptor 2

### Survival

The patients with DND tumors had the highest 5-year overall survival rate (Table 1) (DND 64.7 % vs PND 56.1 % vs D 45.1 %) and were the least likely to have recurrence (DND 27.0 % vs PND 34.3 % vs D 48.3 %). The recurrence patterns varied between the groups. The diffuse tumors were the most likely to have metastatic peritoneal recurrence, whereas systemic recurrence (hematogenous or distant lymph node metastases) was more common in the PND and DND tumors.

**Fig. 2:**
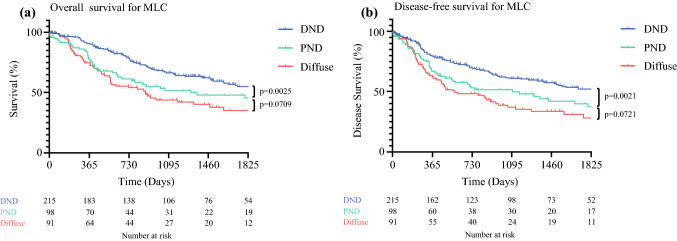
Kaplan-Meier survival analysis of the Tumor Location-Modified Laurén Classification (MLC) for **a** overall survival and **b** disease-free survival. The significance of the difference between survival curves was calculated using the log-rank test

**Fig. 3:**
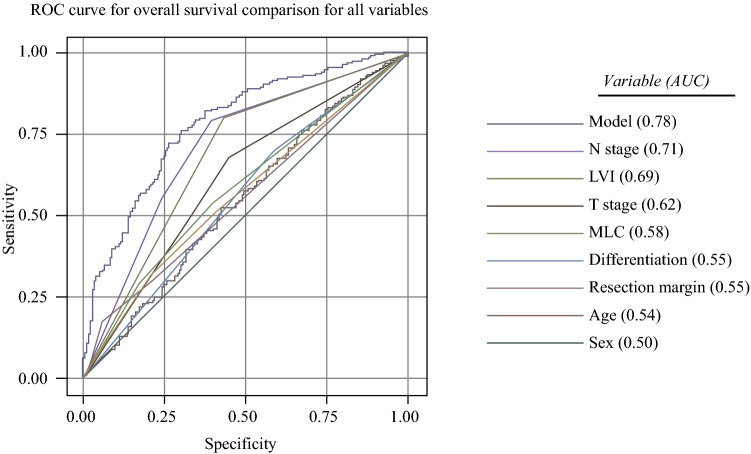
Receiver operating characteristic (ROC) curve analysis for overall survival. Comparison of all variables is listed in order of positive predictive ability

Kaplan-Meier (Fig. 2) and unadjusted Cox regression analysis (Table 3) demonstrated significantly worse overall and disease-free survival for the patients with diffuse and PND tumors. A ROC curve comparison between all variables (Fig. 3) demonstrated the MLC to have a stronger positive predictive ability for overall survival (AUC, 0.58) than differentiation (0.55), resection margin status (0.55), age (0.54), or sex (0.50). Individually, the AUCs for these variables were relatively weak, but the combination of parameters included in the multivariable model provided an AUC of 0.78. A seperate multivariable model including the original Laurén classification instead of the MLC gave a slightly lower AUC value of 0.77.

**Table 3:** Hazard ratios (HR) with 95 % confidence intervals (CI) of overall and disease-free survival for patients who have undergone resection for gastric adenocarcinoma

Variable (*n* = 404)	Unadjusted		Multivariable	
	OS HR 95 % CI	DFS HR 95 % CI	OS HR 95 % CI	DFS HR 95 % CI
Sex				
Male	1.00 (Reference)	1.00 (Reference)	1.00 (Reference)	1.00 (Reference)
Female	1.12 (0.83–1.51)	1.14 (0.84–1.54)	1.09 (0.80–1.48)	1.07 (0.79–1.46)
Age	1.01 (0.99–1.02)	1.01 (0.99–1.02)	1.02 (1.00–1.03)	1.02 (1.00–1.03)
pT stage				
pT0-2	1.00	1.00	1.00	1.00
pT3-4	2.92 (2.12–4.01)	2.84 (2.07–3.91)	1.47 (1.02–2.12)	1.53 (1.06–2.21)
pN stage				
pN0	1.00	1.00	1.00	1.00
pN1	2.76 (1.83–4.19)	2.69 (1.77–4.07)	1.92 (1.25–2.94)	1.97 (1.28–3.01)
pN2-3	4.73 (3.30–6.80)	4.51 (3.14–6.48)	2.53 (1.66–3.84)	2.66 (1.76–4.03)
Differentiation				
Well / moderate	1.00	1.00	1.00	1.00
Poor	1.48 (1.10–2.01)	1.51 (1.12–2.05)	1.05 (0.74–1.49)	1.03 (0.72–1.46)
LVI				
No	1.00	1.00	1.00	1.00
Yes	3.77 (2.63–5.41)	3.62 (2.53–5.19)	2.62 (1.77–3.87)	1.03 (0.72–1.46)
Resection margin				
R0	1.00	1.00	1.00	1.00
R1	2.77 (1.85–4.15)	3.05 (2.04–4.58)	1.54 (0.98–2.43)	1.35 (0.85–2.13)
HER2 status				
Negative	1.00	1.00	N/A	N/A
Positive	0.98 (0.55–1.75)	0.93 (0.52–1.66)		
Modified Laurén classification				
Distal non-diffuse	1.00	1.00	1.00	1.00
Proximal non-diffuse	1.64 (1.16–2.32)	1.68 (1.19–2.39)	1.46 (1.01–2.10)	1.37 (0.95–1.97)
Diffuse	2.20 (1.56–3.09)	2.30 (1.63–3.24)	1.94 (1.29–2.91)	1.89 (1.26–2.84)

*OS* overall survival, *DFS* disease-free survival, *HR* hazard ratio, *CI* confidence interval, *LVI* lymphovascuar invasion; HER2, human epidermal growth factor receptor 2; N/A, Not applicable

Multivariable Cox regression analysis with previously defined covariables (excluding HER2 status due to the presence of missing data and lack of significance in univariate analysis) (Table 3) confirmed PND tumors (HR, 1.46; 95 % CI, 1.01–2.10) and diffuse tumors (HR, 1.94; 95 % CI, 1.29–2.91) to be independent prognostic factors for worse overall survival compared with DND tumors. Other independent factors for worse overall survival were age, advanced pT stage, presence of lymph node metastases, and LVI. Stratified subgroup analysis evaluating neoadjuvant chemotherapy as a potential effect modifier showed similar results both comparatively and to the dataset as a whole.

## Discussion

In this study, the MLC independently predicted survival in a Western cohort of patients with resectable gastric adenocarcinoma. The patients with proximal non-diffuse and diffuse tumors had worse overall survival than those with distal non-diffuse tumors. Much of the previous research into the MLC has been performed on Eastern patients with mixed results.^10, 11^ However, differences in patient characteristics, environmental/dietary factors, tumor biology, and *Helicobacter pylori* positivity have been described between Eastern and Western cohorts.^16, 17^ To our knowledge this is the first study to explore its clinical relevance in a Western population.

Some methodologic issues deserve attention. This study allowed for long-term follow-up evaluation of a large cohort of patients undergoing gastrectomy for gastric cancer. Although data were collected prospectively, the retrospective study design remained susceptible to bias. The observational design made it impossible to rule out confounding despite adjustments for several prognostic factors. As a single-center study, one potential advantage was that all procedures were performed by the same five experienced surgeons, with consistency of multidisciplinary decision-making, thereby reducing heterogeneity. However, this also reduced the generalizability of the findings compared with a population-based approach.

Before evaluating the MLC as a prognostic marker for gastric cancer, it was important to establish where Laurén mixed type tumors were best categorized within this system. Comparison of the clinicopathologic characteristics between Laurén subtypes demonstrated that Laurén mixed tumors were more similar to diffuse-type tumors in some domains (more advanced pT and pN stage, similar low rates of HER2 positivity) and were more comparable with intestinal-type tumors in others (lower margin positivity, higher LVI rates, less tumor recurrence and better survival). Kaplan-Meier analysis suggested that the overall survival of this group more closely resembled the pattern seen in patients with Laurén intestinal-type tumors. For predicting survival, ROC curve analysis demonstrated a slight superiority in categorizing this group with non-diffuse-type tumors. These results suggested that tumors with Laurén mixed-type histology were best placed with non-diffuse tumors in the MLC system when used to predict survival. This is further supported by previous molecular analysis that demonstrated distinct differences in gene expression between non-diffuse and diffuse tumors.^9^ In addition, ROC curve analysis demonstrated a marginal superiority of the MLC over the Laurén classification as a prognostic marker. This supports the findings of a previous study that the MLC had better prognostic discriminatory ability and accuracy than the Laurén classification system.^11^

Although Laurén mixed-type tumors seemed to align more closely with intestinal-type tumors rather than diffuse-type tumors in survival analyses, they appeared to be an intermediate between the two. Considering these results, clinicopathologic differences, the fact that Laurén mixed-type tumors are heterogeneous by definition and that multiple studies have demonstrated different biologic and clinical behaviors in this group,^3, 12–14^ perhaps grouping Laurén mixed-type tumors with either intestinal- or diffuse-type tumors is an oversimplification of a complex issue. A large-scale study is needed to establish whether further molecular or pathologic analysis, such as measurement of the intestinal-type to diffuse-type ratio within Laurén mixed-type tumors, could be better used to classify this group or determine whether they should placed in a separate classification entirely.

It generally is accepted that patients with Laurén diffuse-type tumors have a poorer prognosis.^3–5, 8, 18^ However, the findings of the current study demonstrated that for the patients with Laurén intestinal- or mixed-type histology, proximal tumor location was independently associated with worse survival even after adjustment for tumor stage. This important and clinically relevant finding was not taken into account by the original Laurén classification or tumor-node-metastasis (TNM) staging. Possible explanations include differences in tumor biology, genetic factors, or the increased morbidity associated with total gastrectomy compared with subtotal gastrectomy, although this remains controversial.^19, 20^ Further studies to establish targeted oncologic therapies for this patient group would be of benefit. For example, neoadjuvant chemoradiation has previously demonstrated a survival benefit for locally advanced tumors of the gastric cardia.^21^ However, the benefit compared with systemic chemotherapy remains unproven and is therefore not considered the standard of care in many institutions. Molecular analysis by Shah et al.^9^ demonstrated differences in RNA expression between MLC subtypes. Further analysis to establish how the MLC aligns with other genetic classifications and biomarkers including the Cancer Genome Atlas (TGCA) subtypes, microsatellite instability (MSI), HER2, and programmed death ligand (PDL-1) expression would also be of interest. With emerging research describing tailored treatment based on genetic^22^ and histologic^23, 24^ therapeutic biomarkers and evidence of chemoresistance in patients with diffuse type gastric cancer,^25^ the importance of this is further emphasized.

This study has shown variation in clinical and pathologic characteristics between patients with DND, PND, and diffuse tumors. The patients with diffuse tumors were more likely to be younger and female, a widely accepted association previously described.^3–5^ The patients with PND tumors were more likely to be overweight than those with DND tumors, supporting the findings of numerous previous studies linking obesity with adenocarcinomas of the esophagus and gastroesophageal junction.^26–30^ Both the PND and diffuse tumors were more likely to be locally advanced (pT3–4 and pN+), consistent with previous studies evaluating the MLC.^10, 11^

The diffuse tumors were significantly more likely to have a positive longitudinal resection margin than the DND or PND tumors. Overall, the positive margin rate in the entire cohort was comparable with those of other Western institutions,^31–33^ but the rate for this diffuse subgroup was conspicuously higher. The most likely explanation for this is the presence of microscopic submucosal tumor infiltration seen more commonly in diffuse tumors. Further analysis of the diffuse group showed similar positive margin rates for the patients undergoing subtotal gastrectomy and those undergoing total gastrectomy, with positive proximal and distal margins seen with both operation types. Only a small number of the patients who underwent subtotal gastrectomy had a positive proximal margin, whereas more than half of all the patients with diffuse tumors and positive margins had a positive distal margin. This suggests relatively high rates of duodenal infiltration in this group, a factor not influenced by operation type and more difficult to mitigate. Techniques such as intraoperative frozen section of margins or endoscopic ultrasound to examine the lower esophagus for evidence of infiltration should be considered for this patient group to reduce margin positivity. Although the survival benefit of these techiques remains unproven, any strategy that might improve margin positivity rates should be carefully considered.

In conclusion, this study demonstrated that the Tumor Location-Modified Laurén Classification System was an independent prognostic marker in a Western cohort of patients with resectable gastric adenocarcinoma. The patients with distal non-diffuse tumors had better survival than those with proximal non-diffuse or diffuse tumors.

## Supplementary Information

Below is the link to the electronic supplementary material.

Supplementary file1 (DOCX 18 kb)
