# Supplementary material for: Clinical Relevance of the Tumor Location-Modified Laurén Classification System for Gastric Cancer in a Western Population
Source: Ann Surg Oncol. 2022 Jan 18;29(6):3911–20. doi: 10.1245/s10434-021-11252-y (PMC9072452; doi:10.1245/s10434-021-11252-y)
Supplement: Supplementary file 1 — Supplementary file1 (DOCX 18 kb) [file 10434_2021_11252_MOESM1_ESM.docx]

**Supplementary table 1.** Clinicopathological characteristics comparison between the original Laurén classification.

| **Variable** | | **Intestinal**  *n=229* | **Mixed**  *n=84* | | **Diffuse**  *n=91* | ***p-value*** | |  |
| --- | --- | --- | --- | --- | --- | --- | --- | --- |
| **Age at operation** | | 70 (35-89) | 66 (35-94) | | 65 (27-84) | *Int ^c^ vs Mix ^d^ p=0.007*  *Int vs Dif ^e^ p<0.001*  *Mix vs Dif p=0.52* | |  |
| **Sex** | Male | 168 (73.4%) | 54 (64.3%) | | 48 (52.7%) | *Int vs Mix p=0.12*  *Int vs Dif p<0.001*  *Mix vs Dif p=0.12* | |  |
|  | Female | 61 (26.6%) | 30 (35.7%) | | 53 (47.3%) |  |  |  |
| **BMI** | <25 | 53 (37.3%) | 24 (34.3%) | | 25 (41.0%) | *Int vs Mix p=0.67*  *Int vs Dif p=0.62*  *Mix vs Dif p=0.43* | |  |
|  | ≥25 | 89 (62.7%) | 46 (65.7%) | | 36 (59.0%) |  |  |  |
|  | *Not recorded* | *87* | *14* | | *30* |  |  |  |
| **Neoadjuvant treatment** | Yes | 108 (47.2%) | 45 (53.6%) | | 51 (56.0%) | *Int vs Mix p=0.32*  *Int vs Dif p=0.15*  *Mix vs Dif p=0.74* | |  |
|  | No | 121 (52.8%) | 39 (46.4%) | | 40 (44.0%) |  |  |  |
| **Operation** | Total gastrectomy | 94 (41.0%) | 26 (31.0%) | | 38 (41.8%) | *Int vs Mix p=0.10*  *Int vs Dif p=0.91*  *Mix vs Dif p=0.14* | |  |
|  | Subtotal gastrectomy | 134 (58.6%) | 58 (69.0%) | | 53 (58.2%) |  |  |  |
|  | Other | 1 (0.4%) | 0 (0%) | | 0 (0%) |  |  |  |
| **Recurrence** | No recurrence | 164 (71.6%) | 57 (67.9%) | | 47 (51.6%) | *Int vs Mix p=0.52*  *Int vs Dif p<0.001*  *Mix vs Dif p=0.029* | |  |
|  | Recurrence | 65 (28.4%) | 27 (32.1%) | | 44 (48.4%) |  |  |  |
| **5-year survival** | Alive | 127 (55.5%) | 44 (52.4%) | | 35 (38.5%) | *Int vs Mix p=0.63*  *Int vs Dif p=0.006*  *Mix vs Dif p=0.065* | |  |
|  | Not alive | 102 (44.5%) | 40 (47.6%) | | 56 (61.5%) |  |  |  |
| **Recurrence pattern** | No recurrence | 164 (71.5%) | 57 (68.0%) | | 47 (51.7%) | *Int vs Mix p=0.054*  *Int vs Dif p<0.001*  *Mix vs Dif p=0.058* | |  |
|  | Local recurrence | 5 (2.2%) | 7 (8.3%) | | 6 (6.6%) |  |  |  |
|  | Systemic recurrence ^a^ | 33 (14.3%) | 7 (8.3%) | | 6 (6.6%) |  |  |  |
|  | Peritoneal recurrence | 11 (4.7%) | 7 (8.3%) | | 20 (22.0%) |  |  |  |
|  | Mixed recurrence ^b^ | 17 (7.4%) | 6 (7.1%) | | 12 (13.1%) |  |  |  |
| **pT stage** | pT0-2 | 126 (55.0%) | 26 (31.0%) | 26 (28.6%) | | | *Int vs Mix p<0.001*  *Int vs Dif p<0.001*  *Mix vs Dif p=0.73* | |
|  | pT3-4 | 103 (45.0%) | 58 (69.0%) | 65 (71.4%) | | |  |  |
| **pN stage** | pN0 | 111 (48.5%) | 27 (32.1%) | 29 (31.9%) | | | *Int vs Mix p<0.001*  *Int vs Dif p<0.001*  *Mix vs Dif p=0.79* | |
|  | pN1 | 56 (24.5%) | 10 (11.9%) | 14 (15.4%) | | |  |  |
|  | pN2-3 | 62 (27.1%) | 47 (56.0%) | 48 (52.7%) | | |  |  |
| **Differentiation** | Well / moderate | 144 (62.9%) | 2 (2.4%) | 0 (0%) | | | *Int vs Mix p<0.001*  *Int vs Dif p<0.001*  *Mix vs Dif p=0.14* | |
|  | Poor | 85 (37.1%) | 82 (97.6%) | 91 (100.0%) | | |  |  |
| **Lymphovascular invasion** | No | 85 (37.1%) | 27 (32.1%) | 44 (48.4%) | | | *Int vs Mix p=0.42*  *Int vs Dif p=0.065*  *Mix vs Dif p=0.029* | |
|  | Yes | 144 (62.9%) | 57 (67.9%) | 47 (51.6%) | | |  |  |
| **HER2 status** | Positive | 28 (22.0%) | 5 (7.6%) | 2 (3.6%) | | | *Int vs Mix p=0.011*  *Int vs Dif p<0.001*  *Mix vs Dif p=0.45* | |
|  | Negative | 99 (78.0%) | 61 (92.4%) | 54 (96.4%) | | |  |  |
|  | *Not recorded* | *102* | *18* | *35* | | |  |  |
| **Resection margin** | R0 | 222 (96.9%) | 78 (92.9%) | 66 (72.5%) | | | *Int vs Mix p=0.11*  *Int vs Dif p<0.001*  *Mix vs Dif p<0.001* | |
|  | R1 | 7 (3.1%) | 6 (7.1%) | 25 (27.5%) | | |  |  |

^c^ Int = Intestinal

^d^ Mix = mixed

^e^ Dif = diffuse

**Supplementary Table 2.** Resection margin analysis in patients with diffuse type tumors. Comparison by operation type.

|  | **Subtotal gastrectomy**  *n=53* | **Total gastrectomy**  *n=38* | ***p-value*** |
| --- | --- | --- | --- |
| **R0** | 38 (71.7%) | 28 (73.7%) | *p=0.83* |
| **R1** | 15 (28.3%) | 10 (26.3%) |  |
| *Proximal margin positive* | 3 (5.7%) | 7 (18.4%) | *p=0.08* |
| *Distal margin positive* | 8 (15.1%) | 2 (5.3%) | *p=0.18* |
| *Both margins positive* | 4 (7.5%) | 1 (2.6%) | *p=0.40* |
